# The Fusion of Wide Field Optical Coherence Tomography and AI: Advancing Breast Cancer Surgical Margin Visualization

**DOI:** 10.3390/life13122340

**Published:** 2023-12-14

**Authors:** Yanir Levy, David Rempel, Mark Nguyen, Ali Yassine, Maggie Sanati-Burns, Payal Salgia, Bryant Lim, Sarah L. Butler, Andrew Berkeley, Ersin Bayram

**Affiliations:** 1 Perimeter Medical Imaging AI Inc., 555 Richmond St W #511, Toronto, ON M5V 3B1, Canada; david.rempel@perimetermed.com (D.R.); mburns@perimetermed.com (M.S.-B.); aberkeley@perimetermed.com (A.B.); 2 Perimeter Medical Imaging AI Inc., 8585 N Stemmons Fwy Suite 106N, Dallas, TX 75247, USA; mnguyen@perimetermed.com (M.N.); psalgia@perimetermed.com (P.S.); sbutler@perimetermed.com (S.L.B.); 3 The Edward S. Rogers Sr. Department of Electrical & Computer Engineering, University of Toronto, 27 King’s College Cir, Toronto, ON M5S 1A1, Canada; ali.yassine@mail.utoronto.ca; 4 The Institute of Biomedical Engineering, University of Toronto, 27 King’s College Cir, Toronto, ON M5S 1A1, Canada; bryantby.lim@mail.utoronto.ca

**Keywords:** OCT, optical imaging, AI, breast cancer, deep learning, convolutional neural network, margin visualization, surgical oncology

## Abstract

This study explores the integration of Wide Field Optical Coherence Tomography (WF-OCT) with an AI-driven clinical decision support system, with the goal of enhancing productivity and decision making in breast cancer surgery margin assessment. A computationally efficient convolutional neural network (CNN)-based binary classifier is developed using 585 WF-OCT margin scans from 151 subjects. The CNN model swiftly identifies suspicious areas within margins with an on-device inference time of approximately 10 ms for a 420 × 2400 image. In independent testing on 155 pathology-confirmed margins, including 31 positive margins from 29 patients, the classifier achieved an AUROC of 0.976, a sensitivity of 0.93, and a specificity of 0.98. At the margin level, the deep learning model accurately identified 96.8% of pathology-positive margins. These results highlight the clinical viability of AI-enhanced margin visualization using WF-OCT in breast cancer surgery and its potential to decrease reoperation rates due to residual tumors.

## 1. Introduction

Breast cancer remains the leading cancer-related cause of death among women and is the most common cancer in 109 countries, excluding melanoma, according to the World Health Organization [1]. Early-stage breast cancer management often involves breast-conserving surgery (BCS), a lumpectomy procedure aiming to remove tumors with clear margins while preserving the aesthetic quality of the breast. However, the reliance on permanent histopathology for margin assessment, a process that takes days, results in a significant rate of reoperations due to positive margins. This may necessitate further surgery to remove more tissue, causing increased patient anxiety, higher morbidity, and increased healthcare costs [2]. In a recent 1649 patient study, the margin assessment was performed on 1165 patients (71%), and the overall positive margin rate was 20.8% [3]. Reported rates vary widely from less than 10% to greater than 70% [4,5,6,7,8,9]. The National Surgical Quality Improvement Program (NSQIP) database indicates that post-lumpectomy reoperations are notably higher compared to other organs [10]. Most patients with positive margins undergo a secondary excision operation to reduce the probability of cancer recurrence. These statistics underscore the need for improvements in breast cancer surgery margin assessment.

**Figure 1:**
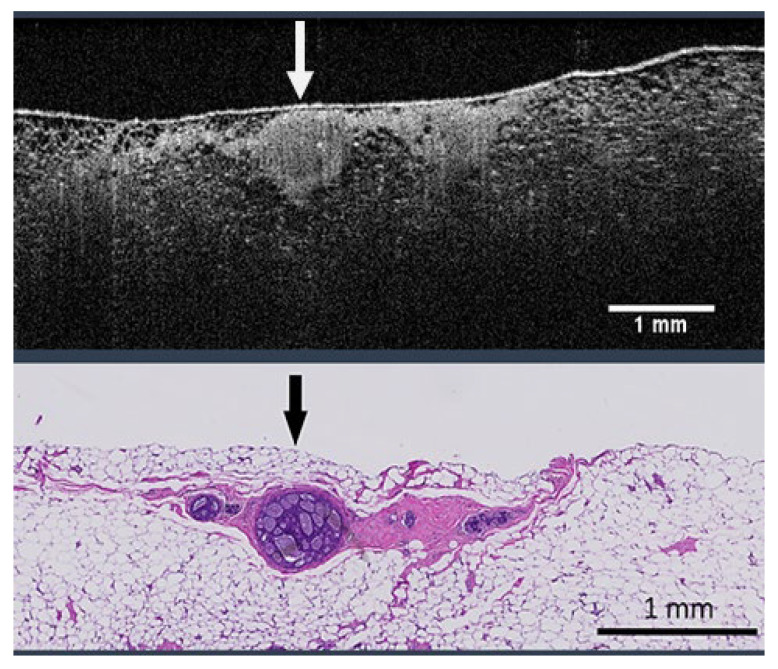
WF-OCT image of breast tissue (**top**) and the corresponding digital pathology image (**bottom**). The arrow in the pathology image points to ductal carcinoma in situ (DCIS), and the same DCIS is clearly visible in the WF-OCT image.

Current intraoperative tumor margin assessment methods include frozen section analysis, imprint cytology, gross assessment, ultrasound imaging, specimen radiography, and optical coherence tomography (OCT). Each has its limitations in accuracy, reporting speed, or both, hampering efficient clinical management. Many surgeons avoid using frozen section analysis for margin management [11] due to its cost and potential interference with permanent histology. Imprint cytology requires on-site expertise, is time consuming, and struggles to detect ductal carcinoma in situ (DCIS) [12]. Gross assessment is often less relevant, as the extent of a lesion might not be clearly discernible [13]. While using ultrasound to guide excision intraoperatively has reduced the rate of positive margins in some studies, it has not shown any difference in positive margin rates for nonpalpable tumors in larger cohort studies [14]. Specimen radiography can help judge the adequacy of excised lesions that show microcalcifications [15], but it has not been proven to reduce reoperation rates for positive margins [16]. OCT offers a promising avenue for real-time, non-invasive, high-resolution imaging to detect malignant breast cancer types, such as invasive ductal carcinoma (IDC) and DCIS [17,18,19]. However, conventional OCT systems, typically used for retinal scanning, offer a limited field of view, making them unsuitable for scanning entire lumpectomy margins [20]. A novel wide-field OCT (WF-OCT) system, designed specifically for intraoperative use in BCS, solves this issue, allowing full breast lumpectomy margin visualization in real-time [21]. This WF-OCT system delivers 10-micron resolution up to a 2 mm imaging depth, which is sufficient to assess BCS margins and significantly higher resolution than specimen radiography or ultrasound. High-resolution images enable correlations to histopathology, allowing histopathological images to serve as the ground truth in AI model training. Figure 1 showcases an exemplary WF-OCT b-scan image of breast tissue (top) with its corresponding histopathology image (bottom). An arrow highlights DCIS in both images, illustrating the ability to identify positive margins. However, any new imaging technology requires clinicians to undergo training to gain confidence. Coupled with the vast amount of imaging data that WF-OCT produces, there is a clear opportunity to employ computer vision and machine learning techniques to streamline the process and boost confidence in using a WF-OCT device in BCS.

The present study details the design and architecture of a WF-OCT deep learning model, evaluates its efficacy in classifying breast tissue, and thoroughly examines its potential in assisting clinicians to mitigate burnout and information overload.

## 2. Materials and Methods

In a regulated industry such as healthcare, adhering to stringent guidelines and best practices is imperative when developing an AI model based on medical imaging data. This study delineates the process of data collection, labeling, training, and testing of the deep learning model. It ensures that the model meets the rigorous criteria required for intra-operative deployment and assistance in surgical decisions.

**Figure 2:**
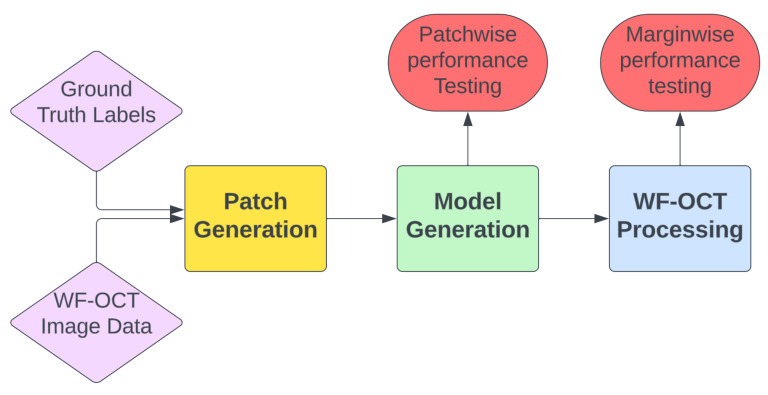
Workflow for model development and performance assessment.

This process encompasses several key steps, starting with the utilization of WF-OCT imaging data, paired with ground truth label sets. Figure 2 presents the overarching workflow for model development and performance assessment. The initial step in our methodology is the strategic splitting of the dataset. We compiled a diverse dataset, which includes a range of disease types and patient demographics. This dataset is then segmented into three parts: training, validation, and an external test set. Our primary focus was to ensure the inclusion of true positive margins—those verified by pathologists and identified as positive in WF-OCT—in each subset. To this end, we employed a manual curation process. Each margin was carefully allocated to ensure that while margins from the same patient could be present in both the training and validation sets, they were completely excluded from the holdout test set, which comprised entirely unique subjects. This approach was mirrored for negative data as well, with each dataset receiving margins from each subject. This method ensures a balanced representation of patient demographics and disease types across all datasets, which is crucial for the robustness of our study. Once the data are partitioned, model development ensues. In the model development pipeline, three distinct tools (patch generation, model generation, and margin processing) are utilized, with the corresponding Python code provided.

1. Patch Generation: In this preliminary step, the ground truth labels are input to extract coordinates from the WF-OCT imaging data. The resulting output consists of labeled image patches, each distinctively named and characterized according to their morphological feature types. These patches are further sorted based on specific margins and unique subject directories. Concentrated data augmentation is implemented to enhance the representation of suspicious features, ensuring a balanced training dataset to the possible extent.
2. Model Generation: This crucial step encompasses both the model training, with specified hyperparameters, and the evaluation of its performance. The model selection emphasizes the epoch exhibiting the lowest validation loss and peak accuracy. Following this, the chosen model undergoes testing using the distinct “test” patches to ascertain key performance metrics and the model’s overall efficacy on a blinded test set.
3. Margin Processing: When a model fulfills the pre-defined performance criteria, it is tested in a simulated real-world environment using the WF-OCT Processing tool. This stage involves the simultaneous processing of designated and complete subject scans as well as the application of a clustering algorithm. The foremost aim is to identify correctly classified key suspicious features and ensure that the model presents the most accurate “Key Thumbnail Images” of the relevant patches to the clinical user. This method boosts user accessibility and efficiency in identifying suspicious features during surgical procedures.

The subsequent sections provide detailed insights into each of these steps and the composition of the training data, as well as the model selection process.

### 2.1. Data Collection and Curation

Our model is designed to offer a swift and accurate assessment of surgical margins. Specifically, it assesses both suspicious and non-suspicious breast morphology through supervised learning. WF-OCT images and their corresponding pathology data were amassed during an IRB-approved clinical trial (Title: “Wide-field optical coherence tomography imaging of excised breast tissue for evaluation of the computer-aided detection tool Imgassist”. IRB #2019-1225) conducted between 2019 and 2021. All participants provided informed consent. The WF-OCT data were partitioned into three distinct sets: the training, validation, and test sets. The first two sets (training and validation, highlighted in Table 1) comprise a total of 585 WF-OCT margin scans from 151 subjects (average age: 63 ± 11.7). It should be noted that some subjects in the training set are not in the validation set and vice versa, which leads to the number of subjects in either set being lower than 151. An independent test set, utilized to benchmark the final model, consisted of 155 margin scans (31 positive and 124 negative) from 29 subjects (average age: 58.5 ± 9.1) with histopathology-confirmed status. A detailed breakdown of the patient demographics, which is proportional to the targeted demographics of model deployment, for the training, validation, and test datasets can be found in Table 1 and Table 2.

The training and testing datasets encompass benign and malignant findings and are listed in Table 2. The inclusion of Table 1 and Table 2 highlights the efforts that were made to include subjects in both training and testing with a variety of benign findings that would pose a challenge to OCT interpretation, including lymphatic invasion, atypical ductal hyperplasia, lobular carcinoma in situ, atypical lobular hyperplasia, usual ductal hyperplasia, and duct ectasia.

**Figure 3:**
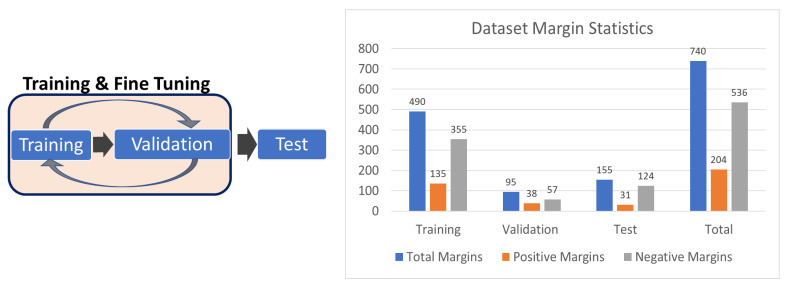
Break-down of the training, validation, and test datasets at margin level along with the total statistics. Training and validation sets are used to train and fine-tune the model, while the test set is blinded to the model for independent performance verification.

For each margin scan, between 200 and 900 WF-OCT images are generated, with the exact number of B-Scan images contingent on the specimen’s size and a user-determined scan density. Figure 3 presents a breakdown of the training, validation, and test datasets, showing margin-level statistics. The figure also delineates the workflow, detailing the specific usage of each dataset during various phases of the training and validation processes. Figure 3 illustrates that the test set is completely independent of the training and validation. The training and testing of the model are limited by the number of positive margins, as there is no shortage of negative margins in the database. The positive margins are separated between training and testing to maximize their utility towards generating a model.

**Figure 4:**
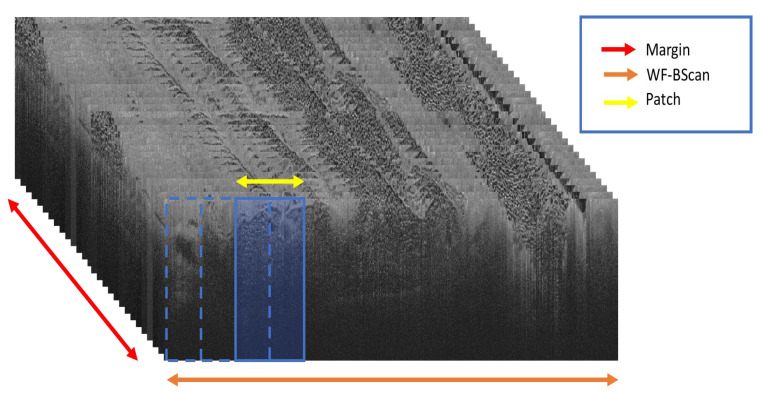
Schematic representation of the WF-OCT imaging framework, showing the hierarchical relationship of the margin (red arrow), composed of sequential WF-Bscans (orange arrows), and a patch (blue box) formed by a sliding window (yellow arrow) over a B-scan.

The model training workflow involved splitting the wide-field OCT (WF-OCT) images into smaller, overlapping patches with a 0.5 step size, each 420 by 188 pixels. Demonstrating the breakdown of the data in a lumpectomy patient is important to understand the full magnitude of information that would be typically reviewed by a clinician and what the model is trained on. A subject would typically have six margins assessed, each margin (formed from a stack of WF-Bscan images) has around 400 b-scans where each WF-Bscan image is divided into overlapping rectangular regions of interest, known as patches, with approximately 30 patches per B-scan image. Ignoring the extra shaves that might have been taken during surgery, this sums up to 72,000 patches for each scan. In our training process, this technique of extracting data was used except for annotated features, where the step size is further reduced to a fifth of the width step to produce an additional five translated patches. Figure 4 provides a visual representation of the relationship between a margin, b-scan, and a patch.

**Figure 5:**
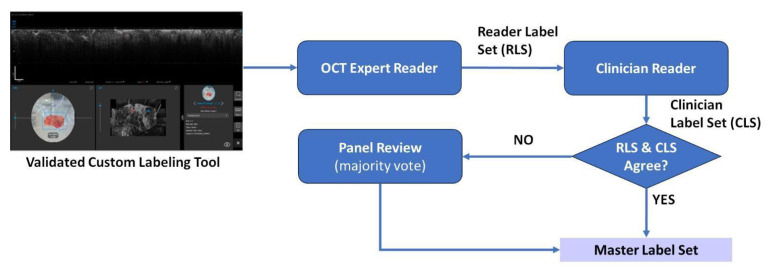
High-level data labeling workflow using a customized validated labeling tool.

To produce the annotated patches, two subject matter experts performed manual annotation of each morphological feature, classifying them as either “suspicious” (malignant) or “non-suspicious” (benign), with the pathology results being the definitive ground truth. First, an expert produces reader label sets using a validated custom labeling tool. This tool allows the reader to select regions of interest with a mouse and assign a label denoting a specific feature type. Additionally, an expert reader uses final pathology to assign a ground truth. The second reader is a clinician with subject matter expertise in the labeled tissue domain. This reader may be a pathologist or breast surgeon. The second reader either agrees or disagrees with the first set of annotations. Additionally, they may add other suspicious region suggestions. Figure 5 provides the high-level workflow of the data labeling process.

### 2.2. Model Development

Convolutional Neural Networks (CNNs) excel in autonomously learning from data, eliminating the need for manually designing image processing pipelines, including filters for specific features [22]. This attribute is especially advantageous in detecting variable lesions in WF-OCT, where lesion characteristics differ among patients. Due to their extensive use in medical imaging and computer vision tasks [23,24], we opted for a CNN-based architecture in our study. In recent years, several deep learning architectures, including ResNet-18 [25], VGG [26], ShuffleNet [27], EfficientNet [28], and MobileNet [29], have gained prominence for their robust performance in medical image classification tasks. These models demonstrate remarkable efficacy in generating accurate ‘disease predictors’, suitable for both binary classification and the nuanced allocation of multi-class disease severity levels. The utility of these architectures is often further enhanced by transfer learning, which allows the models to leverage pre-trained parameters and transfer knowledge from natural images (ImageNet) to the medical imaging domain for more accurate predictions.

However, the current application under investigation presents unique computational constraints that render these conventional models less suitable. Network connectivity, cybersecurity, and data privacy type concerns add additional complexity for cloud-based clinical deployment. Therefore, our system is specifically engineered to classify thousands of image patches in real-time on-edge devices for intraoperative use. This in turn poses additional design constraints due to the inherently limited computational resources available. This constraint mandates innovative approaches to optimizing computational efficiency without compromising the system’s real-time processing and classification accuracy. Traditional architectures, while powerful, are typically designed with a primary focus on achieving state-of-the-art accuracy, often at the expense of increased computational complexity and latency [30]. This complexity manifests as many trainable parameters and floating-point operations per second (FLOPs), both of which are resource-intensive metrics that are not congruent with the real-time, low-latency demands of our application.

**Figure 6:**
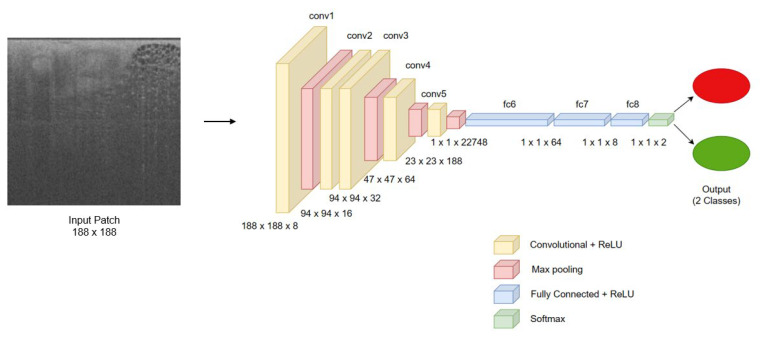
Architecture of the CNN in ImgAssist^TM^.

Our model, specifically designed to remain lightweight to reduce computational burden in the Operation Room (OR), is based on a multi-layered convolutional neural network whose architecture is primarily inspired by the VGG network [26] and other models used for image classification tasks [31]. The final convolutional neural network (CNN) based deep learning (DL) model was crafted to optimize computational efficiency for immediate feedback. It encompasses five convolutional layers (each employing a 3 × 3 kernel) and three fully connected layers, with a cumulative parameter count of approximately 1,589,000. The design of a streamlined architecture for our AI model primarily addresses the challenge of computational resource limitations, with a dedicated Nvidia Quadro RTX 4000 GPU on a device at the time of the model design. This is critical when simultaneously classifying up to 250,000 patches and processing OCT images in real-time on the edge. By optimizing resource efficiency, our model not only manages these concurrent tasks but also aims to significantly reduce time in the OR, a crucial factor in enhancing patient outcomes and operational efficiency. Figure 6 provides an architectural detail of the CNN model (CAUTION—Investigational device. Limited by United States law to investigational use. ImgAssist^TM^ is not available for sale in the United States). 

The ImgAssist CNN model exhibits distinct advantages over conventional architectures like VGG16 and ResNet18 in our specific use case, specifically in computational efficiency. Optimized for 1-channel grayscale images, its compact architecture with significantly fewer parameters (1.5 M compared to 134.2 M for VGG16, 12.6 M for ResNet18, and 3.4 M for MobileNetV2) enhances its suitability for mobile and embedded systems, addressing the limitations of resource-intensive models [29]. ImgAssist also demonstrates reduced computational complexity with lower FLOPs (154 M compared to 15.4 G for VGG16, 1.89 G for ResNet18, and 3.4 M for MobileNetV2), making it less power intensive. Such efficiency is vital for real-time image classification [32]. Moreover, its simplicity aids adaptability to specific tasks [33], an essential feature in specialized domains. With its reduced size and complexity, ImgAssist utilizes a straightforward training process, particularly advantageous in data-limited scenarios [34]. Its architecture is also well-suited for edge computing applications, where cloud data transfer is impractical [35]. In healthcare, a sector where model transparency and compliance are imperative, ImgAssist’s simpler structure may improve explainability and regulatory adherence [36]. These attributes make ImgAssist a potentially more appropriate choice for this specific image classification task than larger, more complex models [37].

### 2.3. Model Performance Assessment in a Clinical Simulation

To evaluate the model’s performance, we implemented an advanced testing methodology that simulates real-world clinical scenarios for disease identification using our margin processing tool. Utilizing the test cohort detailed in the preceding sections, we processed 155 full margins from 29 patients, which included 31 margins flagged as suspicious. This processing was executed in a controlled setting analogous to our WF-OCT device operations, employing a Quadro RTX 4000 GPU for computational support. These margins comprised a total of 1,835,905 image patches, among which 551 were labeled as positive, representing unaugmented, singular patches and constituting a mere 0.03% of the total patch count. We conducted concurrent inference of these patches, assessing both individual and aggregated metrics such as processing time, and margin as well as subject-level accuracy.

#### 2.3.1. Clustering Algorithm Integration for Enhanced Diagnostic Precision

To augment the model’s diagnostic acumen, we incorporated a clustering algorithm using DBSCAN, a density-based, non-parametric clustering technique [38]. This algorithm identifies points in close proximity to each other to form clusters while designating isolated points in sparse areas as outliers. In our application, DBSCAN was employed to cluster adjacent suspicious feature B-scans based on shared x-coordinate values, thus aligning successive patches along the z-axis. We defined a “cluster” as a collection of at least two adjacent detections, which, given the morphological traits of Ductal Carcinoma In situ (DCIS) and Invasive Ductal Carcinoma (IDC), aligns with the expected pattern of manifestations at our chosen patch density. Consequently, this clustering approach shifted our analysis from isolated patches to “Clusters”, enhancing the spatial representation of suspicious areas in WF-OCT scans.

#### 2.3.2. Key Thumbnail Selection for Clinician Review

**Figure 7:**
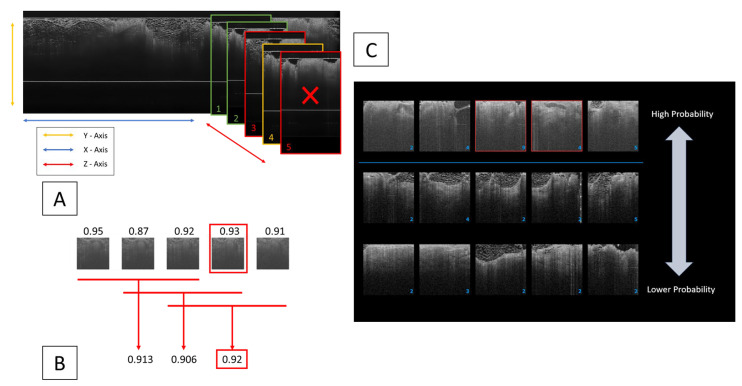
A composite diagram illustrating the multifaceted image analysis process: (**A**) demonstrates the clustering algorithm, retaining only adjacent patches (green) exceeding a set classification threshold, non-suspicious (red) or single (yellow) patches are discarded. (**B**) Details the selection of a ‘Key Thumbnail’ using a moving average maximum (MA_MAX_) method, which identifies the top three contiguous patches with the highest average probability in a cluster; the patch with the maximum local value within this subset is then designated as the ‘Key Thumbnail’. (**C**) Displays the Thumbnail Display Page on the OCT device’s user interface (UI), where clusters with higher confidence are prioritized at the top. ‘Key Thumbnails’ serve as the most representative image of a cluster, providing clinicians with a concise ‘highlight reel’ of suspicious areas within a margin, thereby streamlining the review process, minimizing information overload, and reducing clinician fatigue.

The subsequent phase involved determining the most representative “Key Thumbnail” for clinician review. We calculated this using a moving average maximum (MA_MAX_) algorithm applied to 188 × 188 resized patches within a cluster. This selection algorithm is specifically tailored for clusters larger than two to three thumbnails, addressing cases where clusters could exceed 30 thumbnails, and where a simple midpoint or maximum value selection does not accurately represent the cluster. The “Key Thumbnail” displayed on the device’s user interface (UI) is thus chosen for its highest confidence rating within a significant cluster. Figure 7 illustrates the clustering algorithm, the thumbnail selection process, and the Thumbnail Display Page on the UI.

## 3. Results

We present the evaluation results of the WF-OCT system when enhanced with our deep learning model. This assessment seeks to determine the model’s suitability for practical clinical applications, specifically in the domain of breast cancer surgery margin assessment. Our analysis leverages several performance metrics established by ISO/IEC TS 4213:2022 [39].

### 3.1. Patch-Wise Performance

**Figure 8:**
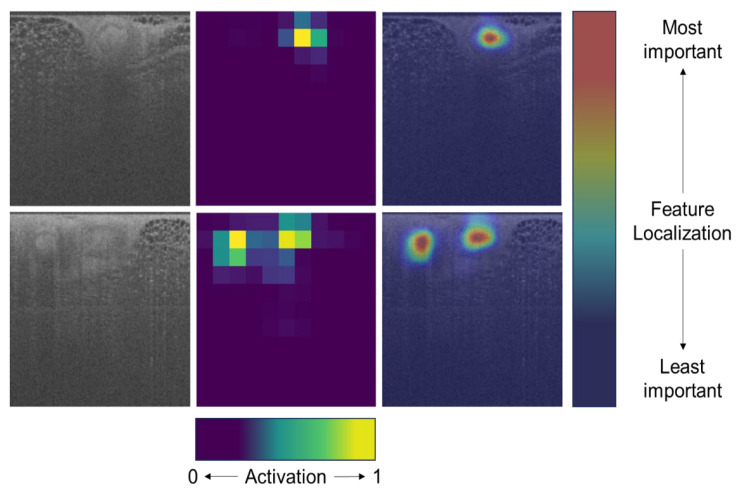
The suspicious thumbnail image on the left is followed by the gradient-weighted Class Activation Maps, which uses the global average of the gradients flowing into the feature maps of the last convolutional layer, a measure that focuses on which features in the image are contributing to the model prediction. The accompanying heatmap overlay on the right provides transparency to the model’s decision making.

Utilizing a comprehensive blinded test set, the convolutional neural network (CNN) model registered an Area Under Receiver Operating Characteristic curve (AUROC) value of 0.976. This indicates a good generalization ability of the model. Given our patch-wise test dataset’s imbalance where only 1.5% (3736 out of 255,682) were positive patches, a baseline AUPRC of 0.146 was established. Against this backdrop, the model’s Area Under Precision–Recall curve (AUPRC) of 0.812 is notably significant, suggesting its robustness in classifying patches. Figure 8 represents an interpretation of the model’s localization capabilities in detecting suspicious features in test patches. These heatmaps, generated by Gradient-weighted Class Activation Mapping (Grad-CAM), indicate which regions in the input image contribute the most to a model’s predictions.

### 3.2. Two-Tiered Confidence Threshold Analysis

Our two-tiered confidence threshold approach is specially crafted to offer clinicians a balanced view of sensitivity and specificity. The classification confidence intervals are found in the performance summary in Table 3. A lower threshold value provides better sensitivity, but it comes at the expense of precision, as evidenced by a lower Positive Predictive Value (PPV) and Matthew’s Correlation Coefficient (MCC). *MCC* is quite useful in binary classification problems as it summarizes the confusion matrix by incorporating True Positives (*TP*), False Positives (*FP*), True Negatives (*TN*), and False Negatives (*FN*) in one single metric (Equation (1)). In the absence of a single threshold that performs well across all performance indicators, we opted for a dual-threshold setting (0.75 and 0.925) to tier the findings. The first threshold is set to be highly sensitive, while the second threshold (0.925) becomes more selective and displays only the higher confidence findings (Precision jumps from 0.41 to 0.79; MCC increases from 0.61 to 0.74 when a higher confidence threshold of 0.925 is used versus 0.75) while trading off to a lower sensitivity performance. This tiered approach provides flexibility to the end user to balance the performance design tradeoffs. A Positive Likelihood Ratio (PLR) of 234.00 at a 0.925 threshold indicates a strong confirmatory value for positive test results. Similarly, a Negative Likelihood Ratio (NLR) of 0.07 at the 0.75 threshold suggests that a negative result is highly indicative of disease absence. These ratios affirm the test’s precision in guiding post-lumpectomy clinical decisions.

(1) $$MCC=\frac{TN\times TP-FN\times FP}{\sqrt{\left(TP+FP)(TP+FN)(TN+FP)(TN+FN\right)}}$$

First Confidence Interval (0.75)

The confidence threshold set at 0.75 yielded the following results:

- Sensitivity: 0.93
- Specificity: 0.98
- Precision (PPV): 0.41
- F1-Score: 0.78
- MCC: 0.61

Second Confidence Interval (0.925)

The metrics achieved at this higher confidence level are as follows:

- Sensitivity: 0.7
- Specificity: 1.0
- Precision (PPV): 0.79
- F1-Score: 0.87
- MCC: 0.74

### 3.3. Margin-Wise Analysis

Our evaluation delves further into assessing the model’s efficacy at full patient margins, termed “clusters”. Additionally, we introduce the concept of “key thumbnails” that are essential for clinician interpretation. Table 4 breaks down the full performance results of each of the chosen confidence intervals, which is followed by a closer look at the cluster-level performance at the margin.

First Confidence Interval Patch-wise Results:

- Evaluated Margins: 155 (31 positive)
- True Positives: 507 (92%)
- True Negatives: 1,894,239 (97.3%)
- False Positives: 53,225 (2.7%)
- Average Positive Patches per Margin: 347 (Positive margins: 882, Negative margins: 213)

Second Confidence Interval Patch-wise Results:

- Evaluated Margins: 155 (31 positive)
- True Positives: 387 (70.2%)
- True Negatives: 1,825,709 (99.5%)
- False Positives: 9645 (0.5%)
- Average Positive Patches per Margin: 65 (Positive margins: 197, Negative margins: 32)

The performance evaluation of the ImgAssist model using two confidence thresholds revealed significant findings. With the first threshold set at 0.75, the model identified positive features within margins with high accuracy, resulting in 30 margins with positive clusters and 27 with positive key thumbnails out of 31 evaluated. The true positive patch detection rate stood at an impressive 92.0%, while the false positive rate was contained at 2.7%. On the other hand, the second threshold at 0.925 demonstrated a slightly reduced true positive rate of 70.2% but substantially minimized false positives to 0.5%, reflecting its precision in distinguishing relevant features. The model effectively discarded single patches that were unlikely to represent disease, indicating an intelligent filtering mechanism. The clustering algorithm proved to be instrumental in reducing the noise from single-patch detections. At the first confidence threshold, 10 single true positive patches were discarded, which, while slightly lowering sensitivity, significantly reduced the potential for false positive distractions. The second confidence threshold saw an increase in discarded single true positives to 42, which aligns with the model’s emphasis on specificity at this level.

The average number of clusters per margin presented interesting insights. At the first threshold, there was an average of 59 clusters per margin, with 147 clusters on average in positive margins, indicating a thorough search for suspicious areas. The second threshold demonstrated a more selective approach, with averages of 10 clusters per margin and 33 per positive margin, pointing to a more focused analysis. Our model processed 155 margins, equivalent to around 1.9 million patches, in a total time of 1504.1 s. This equates to an average of approximately 10.51 s per margin, with a standard deviation of 6.48 s, demonstrating efficient performance suitable for clinical application without significantly extending OR time.

## 4. Discussion

### 4.1. Interpreting Patch-Wise Results

The convolutional neural network (CNN) model’s AUROC value of 0.976 on the blinded test set signifies its strong ability to differentiate between positive and negative patches. An AUROC value close to 1 denotes a model’s excellent discrimination power. As guided by ISO/IEC TS 4213:2022 [39], we also considered the AUPRC due to the high level of data imbalance in the test set. An AUPRC of 0.812, significantly above the baseline of 0.146, underscores the model’s robust performance, especially in prioritizing the positive class. Such high performance in the presence of data imbalance is particularly encouraging, suggesting the model’s resilience against skewed data distributions, a common challenge in medical imaging datasets.

### 4.2. Two-Tiered Confidence Threshold, Patch-Wise Performance

The introduction of a specialized two-tiered confidence threshold illustrates the system’s versatility. Such an approach is instrumental in allowing clinicians to fine-tune their diagnosis based on the desired balance between sensitivity and specificity. This flexibility can be pivotal in diverse clinical scenarios, depending on the level of caution desired.

The sensitivity value of 0.93 at the first confidence interval (0.75) underscores the model’s proficiency in capturing most true positive cases, which is paramount in a medical setting. This is because overlooking a positive case (false negative) can lead to potential clinical oversights, which can have severe repercussions. A specificity of 0.98 also ensures that the model commits minimal errors in identifying negative cases. However, a precision of 0.41 suggests that the model, while erring on the side of caution, might lead to several false alarms. This tradeoff, capturing many true positives while still showcasing an excess number of false positives or “false alarms” indicates that this first confidence interval acts as a sort of “catch-all” being conservative in its approach and adding an additional level of risk mitigation while in use by a clinician. The second confidence interval (0.925) appears to be more exclusionary, prioritizing the minimization of false positives. With a dramatic increase in precision to 0.79 and a specificity of 1.0, this threshold setting might be better suited for situations where reducing false alarms is crucial. However, the tradeoff is evident with a decrease in sensitivity to 0.7. This nuanced approach, balancing specificity and sensitivity, showcases the potential of AI in adapting to varied clinical requirements.

### 4.3. Enhancing Clinical Decision-Making: Integrating AI Model and User Interface for Optimal Margin Performance

This tiered approach, in practice, would allow a clinician to view the highest probability “suspicious” areas first, followed by the lower probability features in case there is no clear indication of disease. Figure 7C provides an example clinical scenario to showcase the user interface (UI) with the two-tier threshold, image clusters, and key thumbnail images built in.

The emphasis on evaluating the model’s efficacy on full patient margins, or “clusters”, accentuates its applicability in real clinical settings. The elimination of isolated single-patch detections is informed by the inherent characteristics of OCT imaging and the typical morphological patterns of DCIS and IDC. By focusing on clusters, the model optimally leverages OCT’s volumetric imaging properties to reduce false positives without compromising on the true positives. This approach signifies a profound understanding of the clinical context in which the AI system operates and provides a novel approach to enhancing the effectiveness of deep learning models using standard clinical practices. Furthermore, the concept of “key thumbnails” facilitates quick clinical assessment, a crucial feature given the time-sensitive nature of clinical decisions. By selecting the most “suspicious” image based on probability metrics, the model aids clinicians in swiftly pinpointing potential areas of concern.

One of the key metrics that emphasize the effectiveness of the system, in the context of UI in a clinical setting, is the average number of clusters per margin, and even more evidently, the ratio of the average number of clusters in a positive margin compared to the number of clusters in a negative margin, 4:1 and 8.25:1, respectively, for the first and second confidence intervals. This ratio provides a clear first indication at the time of surgery whether a margin is more or less likely to contain a suspicious feature and, hence, requires additional action. With 87% and 84% (first and second intervals, respectively) containing the most evident key thumbnail images, a surgeon does not only rely on the reduced number of detections but also can quickly focus on the suspicious areas.

The clinical application capabilities of the proposed classification and implementation framework can be emphasized by highlighting its time-effectiveness in processing margins. With a test dataset composed of 29 subjects (155 margins equivalent to 1.9 million patches), the total scan time recorded in a device-comparable environment is 1504.1 s. This translates to an average processing time of approximately 10.51 s per margin, with a standard deviation of 6.48 s. This efficient processing capability demonstrates the model’s clinical utility and applicability, as it ensures no significant additional time is required in the operating room (OR). Furthermore, the feasibility of running this model in parallel with Wide Field OCT image acquisition on our device further reinforces its practicality, allowing for seamless integration into clinical workflows without disrupting existing OR procedures. This combination of speed and efficiency underscores the potential of ImgAssist in enhancing clinical decision-making processes, offering timely and relevant insights without imposing undue time burdens in critical medical settings.

Upon comparing the performance of the two confidence intervals, their distinct advantages become clear. Together, they equip clinicians with a versatile tool that effectively reduces the risk of overlooking suspicious features, while simultaneously enhancing the efficiency of the image review process. Overall, the model displayed robust performance with the ability to reduce the workload for clinicians by presenting a concise overview of suspicious areas, thereby streamlining the review process and potentially reducing clinician fatigue. The results suggest that the ImgAssist model, particularly with its higher confidence threshold, could significantly contribute to the efficiency and accuracy of disease identification in a clinical setting.

### 4.4. Generalizability and Future Work

#### 4.4.1. Generalizability

The application of Wide-Field Optical Coherence Tomography (WF-OCT) imaging technology transcends beyond breast tissue imaging, presenting a viable approach for surgical oncology margin assessment in various other tissues, provided the OCT image depth of penetration adequately encompasses the pertinent area. The crucial consideration for its extension to alternate tissue indications lies in procuring pathology-correlated WF-OCT data to facilitate the training and optimization of a task-specific AI model. Given that the breast AI model is already informed by WF-OCT images, leveraging transfer learning could offer a more advantageous starting point, consequently reducing data dependence for alternate tissue indications as opposed to constructing a model from the ground up. We tested this hypothesis using existing breast data, comparing the construction of a model from inception against employing transfer learning on EfficientNet; the latter achieved comparable performance utilizing merely 25% of the data needed for a Convolutional Neural Network (CNN) model built de novo, though it did necessitate approximately 4× longer inferencing times.

Another consideration in building training data is the need for fresh tissue specimen imaging with WF-OCT before the pathology processing alters the specimen as tissue composition changes in time as it dries out. The inking process generates artifacts in OCT images, resulting in discrepancies between the training data and target application; it is crucial to collect fresh specimen WF-OCT data for AI training.

#### 4.4.2. Future Work

This work demonstrated the proof of concept for margin visualization through WF-OCT, augmented by a deep learning-driven clinical decision support system. The aim is to assist surgeons intraoperatively by offering suspicious feature identification. One limitation of the study is that the presented results are limited to retrospective blinded test results. A prospective trial is needed to demonstrate clinical efficacy. For this purpose, the AI algorithm has recently been integrated into an investigational WF-OCT device that is being evaluated in an ongoing prospective, multicenter, randomized, double-arm trial focused on evaluating its influence on positive margin rates in breast conservation surgery [40,41]. An analysis of the prospective trial results and the feedback from the trial will inform future development work.

## 5. Conclusions

This paper details the meticulous journey of formulating an AI model tailored for real-time applications in clinical settings, particularly focusing on breast cancer surgery margin assessment, with numerous noteworthy strides being taken. The prudent engineering of the model was a key design constraint, embedding computational efficiency to align with the instantaneous and constrained computational resources typical of surgical environments. Its evaluation, adhering to the rigorous metrics and standards established by ISO/IEC TS 4213:2022 [39], unveiled the model’s discriminative capabilities even amidst dataset imbalances, resulting in an AUROC of 0.976 and AUPRC of 0.812. From a clinical perspective, the deep learning model accurately identified 96.8% of pathology-positive margins, which suggests the potential to improve reported re-excision rates due to positive margins from around 20% to below the 5% mark. This work is currently part of an active prospective, multicenter trial that is randomized and double-armed, with the focus centered on examining its impact on positive margin rates during breast-conserving surgery.

The employment of a two-tiered confidence threshold, conjuring a balanced view of sensitivity and specificity, augments the model’s versatility and practicality in diverse clinical scenarios. Additionally, the incorporation of Grad-CAM underscores a commitment to model interpretability, ensuring that the bridge between AI-based decision support systems and clinician interpretability is robustly constructed. Moving forward, it becomes imperative to weave further into usability, human interpretability, and trust to drive clinical adoption of such AI-based tools.

## Figures and Tables

**Table 1:** Subject demographics. study cohort is limited to adult female breast cancer patients.

Characteristic	Training and Validation (*n* = 151)	Testing (*n* = 29)
Age, years, mean (SD)	63 (11.7)	58.5 (9.1)
Race, *n* (%)		
White	116 (76.8%)	20 (69%)
Black	18 (11.9%)	6 (20.7%)
Asian	10 (6.6%)	3 (10.3%)
Other	6 (4%)	0 (0%)
Not reported	1 (0.7%)	0 (0%)
**Ethnicity, *n* (%)**		
Hispanic or Latino	29 (19.2%)	7 (24.1%)
Not Hispanic or Latino	121 (80.1%)	22 (75.9%)
Unknown	1 (0.7%)	0 (0%)

**Table 2:** Subject disease type statistics, which include both malignant and benign, cancer precursors, findings.

Characteristic	Training and Validation (*n* = 151)	Testing (*n* = 29)
**Malignant Tumor type, *n* (%)**		
Invasive Ductal	27 (17.9%)	8 (27.6%)
Invasive Lobular	4 (2.6%)	0 (0%)
Ductal carcinoma in situ	34 (22.5%)	5 (17.2%)
Mixed	77 (51%)	15 (51.7%)
Benign (Not applicable for tumor type)	5 (3.3%)	1 (3.4%)
**Other findings, *n* (%)**		
Lymphatic invasion	6 (4.0%)	1 (3.4%)
Atypical ductal hyperplasia	23 (15.2%)	7 (24.1%)
Lobular carcinoma in situ	16 (10.6%)	3 (10.3%)
Atypical lobular hyperplasia	15 (9.9%)	9 (31%)
Usual ductal hyperplasia	26 (17.2%)	12 (41.4%)
Duct Ectasia	3 (2.0%)	6 (20.7%)

**Table 3:** CNN Model’s performance parameters across different binary classification thresholds of suspicious findings using independent test data. MCC: Matthew’s Correlation Coefficient, NPV: Negative Predictive Value, PPV: Positive Predictive Value, and LR: Likelihood ratio.

Classification Threshold	Sensitivity (Recall)	Specificity	F1-Score	Matthew’s Correlation Coefficient (MCC)	Positive Predictive Value (PPV) (Precision)	Negative Predictive Value (NPV)	Positive Likelihood Ratio	Negative Likelihood Ratio
0.5	0.96	0.969	0.73	0.542	0.317	0.999	30.97	0.04
0.6	0.948	0.974	0.749	0.567	0.35	0.999	36.46	0.05
0.7	0.935	0.978	0.768	0.594	0.387	0.999	42.50	0.07
0.75	0.928	0.98	0.779	0.609	0.41	0.999	46.40	0.07
0.8	0.894	0.986	0.808	0.648	0.479	0.998	63.86	0.11
0.9	0.768	0.996	0.871	0.743	0.727	0.997	192.00	0.23
0.925	0.702	0.997	0.868	0.737	0.782	0.996	234.00	0.30
1	0	1	0	0	1	0	-	1.00

**Table 4:** Margin level performance statistics of simulated clinical test cases at 2 confidence thresholds.

Metric	1st Confidence Threshold (0.75)	2nd Confidence Threshold (0.925)
Number of Margins Evaluated	155	155
Number of Positive Margins	31	31
Positive Identification (Margins with Clusters/Key Thumbnails)	30/27	26/26
True Positive Patches (%)	507 (92.0%)	387 (70.2%)
False Negative Patches (%)	44 (8.0%)	164 (29.8%)
True Negative Patches (%)	1,894,239 (97.3%)	1,825,709 (99.5%)
False Positive Patches (%)	53,225 (2.7%)	9645 (0.5%)
Average Patches per Margin (Positive/Negative)	882/213	197/32
Discarded Single Patches (True Positive/True Negative)	10/18,629	42/5234
Clusters (Total/with True Positives)	9135/154	1515/103
True Positive Key Thumbnails	91	74
Average Clusters per Margin (Positive/Negative)	147/37	33/4
Scan Times (Seconds) (Total/Average Margin/Std Dev)	1504.1/10.51/6.48	

## Data Availability

Data is contained within the article and supplementary material.

## Supplementary Materials

The packaged Python code for patch generation, labeling, and model generation tool can be downloaded at: https://www.mdpi.com/article/10.3390/life13122340/s1. Please review the README file for further details on the usage and necessary libraries.
